# SLC45A4 promotes glycolysis and prevents AMPK/ULK1‐induced autophagy in TP53 mutant pancreatic ductal adenocarcinoma

**DOI:** 10.1002/jgm.3364

**Published:** 2021-06-02

**Authors:** Wenying Chen, Fengting Huang, Jing Huang, Yuanhua Li, Juanfei Peng, Yanyan Zhuang, Xianxian Huang, Liting Lu, Zhe Zhu, Shineng Zhang

**Affiliations:** ^1^ Department of Gastroenterology, Sun Yat‐Sen Memorial Hospital Sun Yat‐Sen University Guangzhou China; ^2^ Department of Oncology Sun Yat‐Sen Memorial Hospital, Sun Yat‐Sen University Guangzhou China; ^3^ Guangdong Provincial Key Laboratory of Malignant Tumor Epigenetics and Gene Regulation Sun Yat‐Sen Memorial Hospital, Sun Yat‐Sen University Guangzhou China; ^4^ Department of General Surgery Peking University Shenzhen Hospital Shenzhen China; ^5^ Department of Gastroenterology Tungwah Hospital of Sun Yat‐Sen University Dongguan China; ^6^ Center of Digestive Endoscopy The Eighth Affiliated Hospital, Sun Yat‐Sen University Shenzhen China; ^7^ Herbert Irving Comprehensive Cancer Center Columbia University New York NY USA

**Keywords:** AMPK pathway, autophagy, glucose metabolism, SLC45A4, TP53 mutation

## Abstract

**Background:**

Somatic mutations of the TP53 gene occur frequently in pancreatic ductal adenocarcinoma (PDA). Solute carrier family 45 member A4 (SLC45A4) is a H^+^‐dependent sugar cotransporter. The role of SLC45A4 in PDA, especially in TP53 mutant PDA, remains poorly understood.

**Methods:**

We explored the TCGA datasets to identify oncogenes in TP53 mutant PDA. MTS [3‐(4,5‐dimethylthiazol‐2‐yl)‐5‐(3‐carboxymethoxyphenyl)‐2‐(4‐sulfophenyl)‐2H‐tetrazolium], colony formation and 5‐ethynyl‐2′‐deoxyuridine (Edu) assays were performed to investigate the function of SLC45A4 *in vitro*. Glucose consumption, lactate production and ATP production were detected to evaluate glucose utilization. Extracellular acidification rate and oxygen consumption rate assays were used to evaluate glycolysis and oxidative phosphorylation. The subcutaneous xenotransplantation models were conducted to explore the function of SLC45A4 *in vivo*. RNA‐sequencing and gene set enrichment analysis were employed to explore the biological alteration caused by SLC45A4 knockdown. Western blotting was performed to evaluate the activation of glycolysis, as well as the AMPK pathway and autophagy.

**Results:**

SLC45A4 was overexpressed in PDA for which the expression was significantly higher in TP53 mutant PDA than that in wild‐type PDA tissues. Moreover, high level of SLC45A4 expression was tightly associated with poor clinical outcomes in PDA patients. Silencing SLC45A4 inhibited proliferation in TP53 mutant PDA cells. Knockdown of SLC45A4 reduced glucose uptake and ATP production, which led to activation of autophagy via AMPK/ULK1 pathway. Deleting SLC45A4 in TP53 mutant HPAF‐II cells inhibited the growth of xenografts in nude mice.

**Conclusions:**

The present study found that SLC45A4 prevents autophagy via AMPK/ULK1 axis in TP53 mutant PDA, which may be a promising biomarker and therapeutic target in TP53 mutant PDA.

## Graphical abstract

Knockdown of solute carrier family 45 member 4 (SLC45A4) reduced glucose uptake and ATP production, which led to activation of autophagy via the AMPK/ULK1 pathway in TP53 mutant pancreatic ductal adenocarcinoma cells.

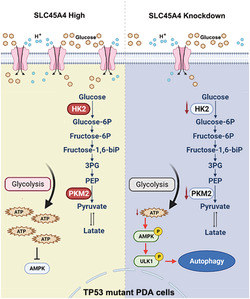

## 1 INTRODUCTION

The pancreatic ductal adenocarcinoma (PDA) is an aggressive disease with high morbidity. TP53 is one of the most frequently mutated genes in PDA.^1^, ^2^ The wild‐type p53 is a tumor suppressor, which transcriptionally activates target genes to invoke anti‐proliferative processes, such as apoptosis, cell cycle arrest and DNA repair.^3^ Intriguingly, the mutant p53 obtains a gain‐of‐function (GOF) activity.^4^, ^5^, ^6^ Recent evidence has demonstrated that mutant p53 increases glucose intake and glycolytic activity, which promotes the Warburg effect in cancer cells.^7^, ^8^, ^9^, ^10^ Targeting the mutant p53 might be an attractive strategy to reprogram glucose metabolism for cancer therapy. However, the downstream genes of mutant p53 or the genes interplay with mutant p53 in regulating cancer metabolism are largely unknown. The identification of oncogenes that specifically accompany mutant p53 in PDA might provide prognostic information and favor the development of more effective targeted therapies.

Solute carrier family 45 (SLC45) members are H^+^‐dependent sugar cotransporters.^11^, ^12^ The four members in this family, A1 to A4, have been confirmed to transport both the monosaccharides and the disaccharide sucrose. SLC45 family members may be involved in glucose metabolism, energy supply, melanin synthesis and osmotic pressure adjustment.^12^, ^13^, ^14^ Emerging evidence has indicated that aberrant expression of SLC45 proteins, such as SLC45A2 and SLC45A3, is associated with tumorigenesis and poor prognosis of cancer patients.^15^, ^16^ However, the role of this family members in PDA, especially in TP53 mutant PDA, remains poorly understood.

In the present study, we revealed that SLC45A4 was the most highly expressed SLC45 family member in PDA and significantly up‐regulated in TP53 mutant PDA. Its overexpression was associated with poor prognosis of PDA patients. Intriguingly, knockdown of SLC45A4 inhibited cell growth, uptake of glucose and production of ATP in TP53 mutant PDA cells, but not in TP53 wild‐type pancreatic duct cells. Decreased energy production in SLC45A4 knockdown cells induced autophagy through the AMPK/ULK1 axis. Together, our results reveal a novel role of SLC45A4 and provide a promising therapeutic strategy for TP53 mutant PDA.

## 2 MATERIALS AND METHODS

### 2.1 Public data collection and bioinformatics analysis

The RNA expression profiles of SLC45 family were obtain from Gene Expression Profiling Interactive Analysis (GEPIA) (http://gepia.cancer-pku.cn/index.html). The data of tumor samples based on the The Cancer Genome Atlas (TCGA) database, whereas that of the normal samples based on the TCGA and the GTEx databases. The protein expression of SLC45 family was obtain from The Human Protein Atlas (HPA) (https://www.proteinatlas.org/). SLC45A4 protein level was evaluated based on its immunohistochemistry (IHC) staining in HPA database. The RNA expression data and TP53 gene mutation data as well as clinical data in PDA patients were assessed using the cBioportal for Cancer Genomics database (https://www.cbioportal.org/) and TCGA (https://tcga-data.nci.nih.gov/tcga/). The survival analysis of SLC45A4 for PDA patients in TCGA profile was conducted in GEPIA, in which quartile was set as the cut‐off value. The TP53 mutation information of PDA cell lines was downloaded from Broad Institute Cancer Cell Line Encyclopedia (CCLE) (https://portals.broadinstitute.org/ccle).

### 2.2 Clinical specimens and ethical approval

A total of paraffin‐embedded 83 PDA tissues and 23 adjacent non‐tumor tissues for immunohistochemistry stain were obtained from patients undergoing surgery at Sun Yat‐Sen Memorial Hospital, Sun Yat‐Sen University (Guangzhou, China). All tissue samples were preserved from January 2018 through July 2019 with histological confirmation. The patients had not received preoperative chemotherapy or radiotherapy. All patients provided their written informed consent. The protocols conducted in the study were approved by the Ethics Committee of Sun Yat‐Sen Memorial Hospital.

### 2.3 Cell culture, transfection and treatment

The PDA cell lines, including BxPC‐3, Capan‐2, HPAF‐II, MIA PaCa‐2, PANC‐1, SW1990 and the immortalized pancreatic ductal epithelial cell line hTERT‐HPNE, were purchased from American Type Culture Collection (ATCC, Rockville, MD, USA). hTERT‐HPNE cells were cultured in glucose‐free DMEM (11966025; Gibco, Gaithersburg, MD, USA) and Medium M3 Base (M300F‐500; Incell Corp., San Antonio, TX, USA) in a ratio of 3:1, supplemented with 5.5mm d‐glucose (G7021; Sigma‐Aldrich, St Louis, MO, USA), 5% fetal bovine serum (16140071; Gibco), 10 ng/ml human recombinant EGF (E9644; Sigma‐Aldrich) and 750 ng/mL puromycin (A1113802; Gibco). The PDA cell lines were cultured in RPMI1640 medium (A1049101; Gibco) supplemented with 10% fetal bovine serum (16140071; Gibco). All cells were incubated at 37°C in a humidified atmosphere containing 5% CO_2_.

The small interfering RNA (siRNA) oligonucleotides specifically targeting SLC45A4 and the negative control (si‐NC) were purchased from RiboBio (Guangzhou, China) with the sequences: SLC45A4: siRNA‐1, 5′‐CCACATTCCTGGTGATCTA‐3′; siRNA‐2, 5′‐GACGAGACCTTGCTGGATA‐3′. Lipofectamine RNAiMAX Transfection Reagent (13778030; Invitrogen, Carlasbad, CA, USA) was adopted for transient transfections in accordance with the manufacturer's instructions.

The SLC45A4 knockout cell lines were generated using the CRISPR/Cas9 system ordered from GENECHEM (Shanghai, China). The SLC45A4 sgRNA (sequence: 5′‐GCACCTGTTCAGCATCGACG‐3′) was cloned into GV392 plasmid. Cells were first infected with Lenti‐CAS9‐puro virus. After 48 h, relative sg‐SLC45A4 GV392 plasmid was transfected and then selected using puromycin (540222; Sigma‐Aldrich) and G418 (A2513; APExbio, Houston, TX, USA). Knockdown efficiency was verified by western blot analysis.

### 2.4 RNA extraction and quantitative real‐time polymerase chain reaction (RT‐PCR)

Total RNA was isolated and extracted using the MolPure® Cell/Tissue Total RNA Kit (19221ES08; Yeason, Shanghai, China) in accordance with the manufacturer's instructions. The concentration and purity of RNA was detected with a NanoDrop Spectrophotometer (Thermo Fisher Scientific, Waltham, MA, USA). Total RNA (500 ng) was reverse transcribed to cDNA in a final volume of 20 μl using HiScript II Q Select RT SuperMix for qPCR (R233‐01; Vazyme, Nanjing, China). Real‐time PCR was performed using ChamQ SYBR qPCR Master Mix (Q311‐02; Vazyme) and analyzed with the Roche LightCycler system (Roche, Basel, Switzerland). Gene expression was standardized to the reference gene β‐actin and calculated using the 2^−∆∆CT^ method.

The primer sequences used were: human SLC45A4, 5′‐AACGTGTCAGAGGAGGCCAA‐3′ (forward) and 5′‐GTAAACGGGAGTGCGGCTCA‐3′ (reverse); human β‐actin, 5′‐TGGAACGGTGAAGGTGACAG‐3′ (forward) and 5′‐AACAACGCATCTCATATTTGGAA‐3′ (reverse).

### 2.5 Western blotting

The cells were washed with ice‐cold phosphate‐buffered saline (PBS) and cells were lysed on ice with RIPA buffer (CW2333; CoWin Biosciences, Beijing, China) containing 1% Phosphatase Inhibitor Cocktail (CW2383; CoWin Biosciences) and Protease Inhibitor Cocktail (P8340; Sigma‐Aldrich). Cell lysates were centrifugated at 12,000 ***g*** for 20 min at 4°C. Aspirated the supernatant and measured the protein concentration using a BCA Protein Assay Kit (CW0014; CoWin Biosciences). Equal amount of protein was separated via 10% or 12% sodium dodecyl sulphate–polyacrylamide gel electrophoresis (CW0022; CoWin Biosciences) and then transferred to a polyvinylidene fluoride membrane (IPVH00010; Millipore, Burlington, MA, USA). After blocking with 5% bovine serum albumin, the membrane was incubated with the primary antibodies overnight at 4°C. Antibodies recognizing SLC45A4 (NBP1‐70394; dilution: 1:200; Novus Biologicals, Littleton, CO, USA), HK2 (2867; dilution 1:1000; Cell Signaling Technology, Danvers, MA, USA), PKM2 (4053; dilution 1:1000; Cell Signaling Technology), p‐AMPK (2535; dilution 1:1000; Cell Signaling Technology), AMPK (ab32047; dilution 1:1000; Abcam, Cambridge, MA, USA), p‐ULK1 (5869; dilution 1:1000; Cell Signaling Technology), ULK1 (ab128859; dilution 1:1000; Abcam), p62 (5114; dilution 1:1000; Cell Signaling Technology), LC3b (ab48394; dilution 1:1000 Abcam) and β‐actin (HRP‐66009; dilution 1:3000; Proteintech, Rosemont, IL, USA). Incubated the membranes with HRP‐conjugated secondary antibodies except β‐actin, and blots were then visualized with by chemiluminescence (ECL Western Blotting Substrate; 32209; Pierce, Rockford, IL, USA) and photographed with the BLT GelView 6000 Pro machine (Biolight, Guangzhou, China) or the G:BOX Chemi XT4 (Syngene, Bangalore, India).

### 2.6 IHC

Tissues samples were harvested in 4% formaldehyde buffered, embedded in paraffin and then sectioned at between 4‐5 μm thickness. Antibodies against SLC45A4 (NBP1‐70394; dilution 1: 200; Novus Biologicals), Ki‐67 (ab15580; dilution 1: 400; Abcam) were used for immunohistochemical analyses. All slides were incubation with primary antibody overnight at 4°C and then incubated with a labeled polymer‐HRP for 1 h at room temperature followed by development with chromogen at room temperature. SLC45A4 IHC scores were calculated from multiplying the proportion of positively stained cells by staining intensity. The proportion of positively stained cells was scored as: 0, none; 1, 1–25%; 2, 26–50%; 3, 51–75%; and 4, more than 75%. Staining intensity was scored as: 0 (negative), no staining; 1 (weak), pale brown; 2 (moderate), brown; 3 (strong), dark brown. All samples were assessed by two evaluators in an independent manner.

### 2.7 RNA‐sequencing (RNA‐seq) and gene set enrichment analysis (GSEA)

Three repeated samples from KO‐SLC45A4 cells and control cells were collected following by total RNA isolation and extraction. The preparation of cDNA library and RNA sequencing was performed by GENECHEM (Shanghai, China).

GSEA was performed to investigate the potential gene sets affected by SLC45A4 knockdown. The Molecular Signatures Database (https://www.gsea-msigdb.org/gsea/msigdb/) was used for running GSEA. Gene sets with *p* < 0.05 and adjusted *q*‐values [false discovery rate (FDR)] < 0.25 were considered enriched between the classes under comparison.

### 2.8 Cell growth assay

Cells transfected with indicated oligo‐nucleotides for 24 h were harvested and dissociated into single‐cell suspensions. For cell proliferation assays, cells were plated at a concentration of 1000 cells/200 μl into a 96‐well plate. Further, the cells were cultured under normal conditions for 5 days and subsequently tested by the MTS [3‐(4,5‐dimethylthiazol‐2‐yl)‐5‐(3‐carboxymethoxyphenyl)‐2‐(4‐sulfophenyl)‐2H‐tetrazolium] assay every day for plotting cell proliferation curves. For the colony formation assay, cells were plated at a density of 1000 cells/2 ml into a six‐well plate. After 10–14 days of incubation, the cells were fixed and stained to count the number of clones. For 5‐ethynyl‐2′‐deoxyuridine (EdU) detection, cells were plated at a density of concentration of 10,000 cells/200 μl onto a 96‐well plate. EdU immunofluorescence staining was carried out with the EdU kit (C10310‐1; RiboBio) and photographed with an IX73 microscope (Olympus, Tokyo, Japan).

### 2.9 Glucose utilization assay, lactate production and intracellular ATP production

PDA cells were transfected transiently with siRNAs when the cells were 60–80% confluent in a six‐well tissue culture plate. For the glucose utilization assay and lactate production, after 12 h, the previous media was replaced with phenol red free RPMI 1640 media supplemented with 1% fetal bovine serum and cultured for 48 h. The glucose concentration was measured with a colorimetric glucose detection kit (EIAGLUC; Thermo Fisher Scientific). The amounts of lactate production were assessed using the lactate assay kit (MAK064; Sigma‐Aldrich) in accordance with the manufacturer's instructions. The amounts of ATP production were quantified using an ATP assay kit (S0026; Beyotime, Shanghai, China) in accordance with the manufacturer's instructions. The protein concentration was measured as described above and the normalized ATP level was calculated via the protein concentration.

### 2.10 Extracellular acidification rate and oxygen consumption rate assays

The oxygen consumption rates (OCR) and the extracellular acidification rates (ECAR) were measured using Glycolysis Stress Test Kit (103344‐100; Agilent Technologies, Santa Clara, CA, USA) and a Seahorse XF Cell MitoStress Test Kit (103575‐100; Agilent Technologies) on the Seahorse XFe96 Extracellular Flux Analyzer (Seahorse Biosciences, North Billerica, MA, USA) in accordance with the manufacturer’s instructions. Briefly, after transient transfection with siRNAs or negative control, 1.5 × 10^4^ hTERT‐HPNE cells, 1.5 × 10^4^ HPAF‐II cells and 2 × 10^4^ MIA PaCa‐2 cells were respectively seeded onto a 96‐well plate followed by overnight incubation. After baseline measurements, for ECAR, 100 mm glucose, 10 μm oligomycin and 500 mm 2‐deoxy‐glucose were automatically and successively injected to each well. For OCR, 15 μm oligomycin, 10 μm FCCP (carbonyl cyanide‐p‐trifluoromethoxyphenylhydrazone) and 5 μm rotenone/antimycin A were injected to each well. The ECAR and OCR values were calculated by normalization to the protein concentration.

### 2.11 Transmission electron microscopy (TEM)

TEM was performed as previously described.^17^ Briefly, cells were collected by centrifugation to obtain a pellet with a size in the range 10–30 μl. Then, the cell pellet was fixed with 2.5% glutaraldehyde followed by postfixing in 1% osmium tetroxide, staining in 2% uranyl acetate, dehydrating and infiltrating with resin. The number of autophagic vacuoles was counted for autophagic profiles per cell area on sections with uniform random sampling, and the images were photographed with a JEM‐100CX‐II transmission electron microscope (Joel, Tokyo, Japan).

### 2.12 Subcutaneous xenotransplantation models

The animal experiment was conducted after receiving approval from the Research Animal Resource Center of Sun Yat‐Sen University. Female BALB/C nude mice at age of 5 weeks (*n* = 5 per group) were subcutaneously injected at 2 × 10^6^ cells (resuspended in 100 μl of PBS solution) per mouse into the back flank. Tumor growth was assessed using calipers weekly for 4 weeks. The tumor volume was determined as length × width^2^ × 0.5.

Animal studies were approved by the Institutional Animal Care and Use Committee of Sun Yat‐sen University, Guangzhou, China (protocol code 180213).

### 2.13 Statistical analysis

All continuous variables were represented as the mean ± SD. Categorical variables were presented as percentages. Non‐parametric variables were compared by the chi‐squared test. For parametric variables, a two‐tailed Student's *t* test was used to indicate differences between two groups or one‐way analysis of variance was used for more than two groups. Kaplan–Meier analysis and a log‐rank test were used for survival analysis. *p* < 0.05 was considered statistically significant.

## 3 RESULTS

### 3.1 Expression of SLC45A4 is associated with TP53 mutation status and poor clinical outcomes of PDA patients

**FIGURE 1:**
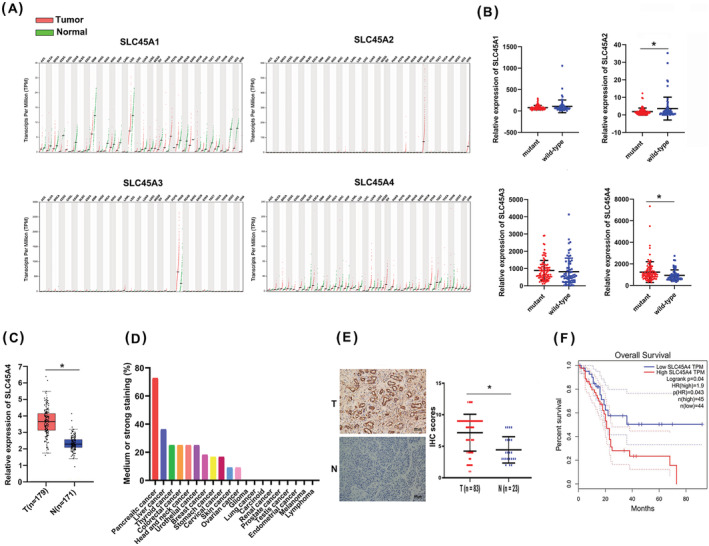
Expression of SLC45A4 is up‐regulated in PDA and associated with poor clinical outcomes. (A) Expression of SLC45 family members in TCGA datasets was analyzed via the GEPIA web server. (B) Expression of SLC45 family in TP53 wild‐type or mutant PDA based on TCGA profile. (C) SLC45A4 expression was higher in PDA tissues based on TCGA profile. (D) SLC45A4 protein expression in various cancers based on the HPA database. (E) IHC showed the higher SLC45A4 expression level in PDA tissues compared to normal tissues. Scale bar = 200 μm. (F) Kaplan–Meier survival analysis of overall survival in TCGA PDA cases using the GEPIA server. *p < 0.05, **p < 0.01 and ***p < 0.001

We investigated the gene expression of SLC45 family members in TCGA datasets from the GEPIA web server,^18^ which showed that SLC45 family members, especially SLC45A4, were overexpressed in many types of cancer (Figure 1A). Given that TP53 is one of most frequently mutated genes in PDA, to determine whether the expression of SLC45 family members was associated with TP53 status, we analyzed the TCGA PDA dataset. Among the SLC45 family, the mRNA level of SLC45A4 in TP53 mutant group was significantly higher than that in TP53 wild‐type group, whereas the other three members did not show significant results (Figure 1B).

Then we confirmed that the mRNA expression of SLC45A4 was significantly higher in PDA than that in normal tissues in the TCGA and GTEx dataset (Figure 1C). The IHC staining data from the HPA database also showed that SLC45A4 protein was highly expressed in PDA tissues (Figure 1D). Moreover, in our clinical samples, higher expression of SLC45A4 was observed in PDA tissues than in normal tissues (Figure 1E). Statistical analysis revealed that SLC45A4 expression was associated with the CEA level and lymph node stage (Table 1). Importantly, PDA tissues with higher SLC45A4 expression had significant worse overall survival of patients (hazard ratio = 1.9, *P* = 0.043) (Figure 1F). These data showed that SLC45A4 expression was significantly up‐regulated in PDA tissues and its high expression was associated with a poor prognosis of PDA patients.

**TABLE 1:** Clinicopathological characteristics of 83 PDA patients

Characteristics	Total (*n* = 83)	SLC45A4		*p* value
		High (*n* = 52)	Low (*n* = 31)	
Gender				0.396
Male	53	35	18	
Female	30	17	13	
Age (years)				0.934
≥ 60	46	29	17	
< 60	37	23	14	
Location				0.304
Head	64	42	22	
Body or tail	19	10	9	
Histologic grade				0.363
Well	7	6	1	
Moderately or poorly	76	46	30	
CEA				0.003*
High	36	29	7	
Normal	47	23	24	
CA199				0.082
High	67	45	22	
Normal	16	7	9	
pT stage				0.178
pT1 + pT2	54	31	23	
pT3 + pT4	29	21	8	
pN stage				0.047*
pN0	34	17	17	
pN1 + pN2	49	35	14	
pM stage				1
pM0	79	49	30	
pM1	4	3	1	
Liver metastasis				1
Absent	79	49	30	
Present	4	3	1	

### 3.2 SLC45A4 knockdown inhibits the proliferation of TP53 mutant PDA cells *in vitro* and *in vivo*

**FIGURE 2:**
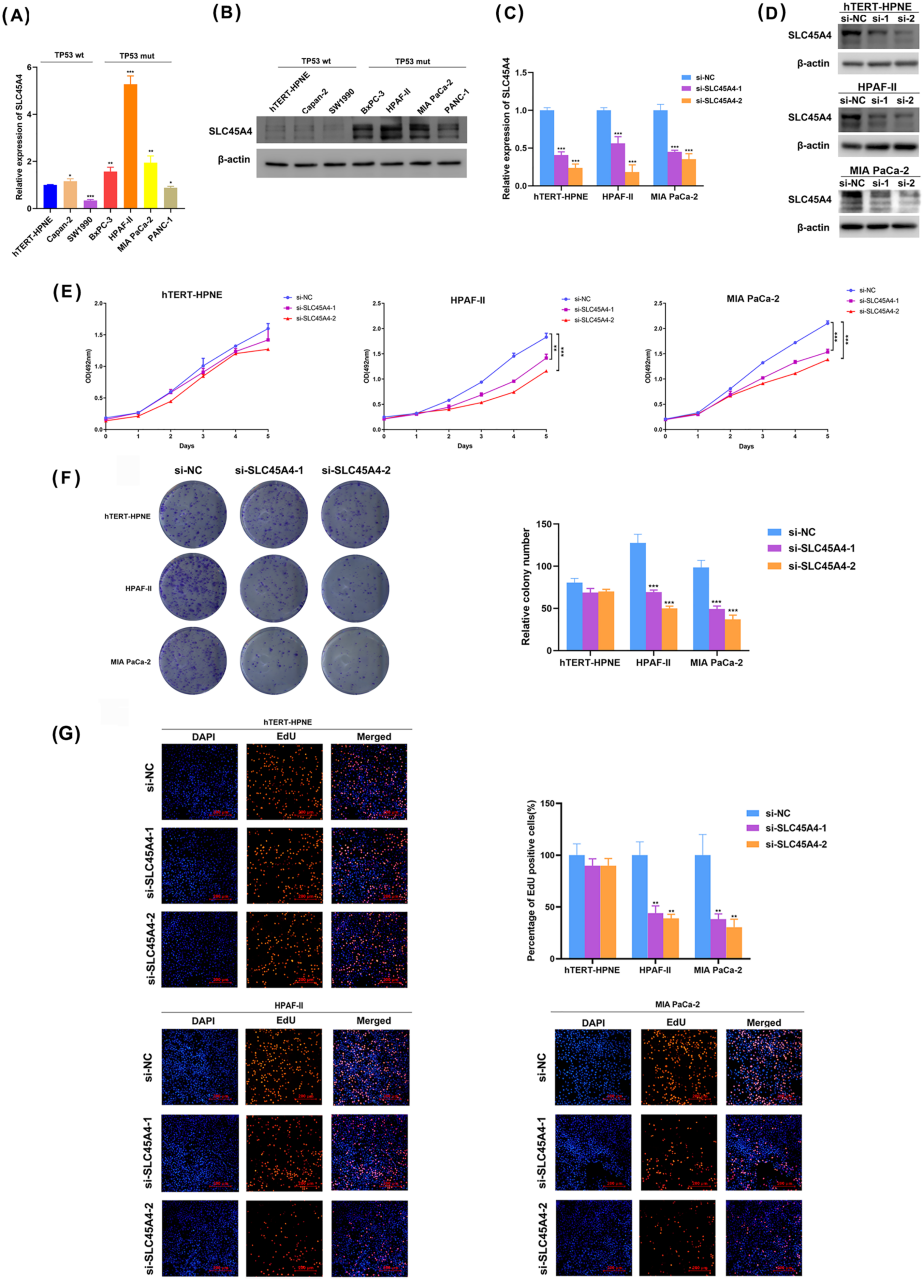
Knockdown of SLC45A4 inhibits proliferation in TP53 mutant PDA cells *in vitro*. qRT‐PCR (A) and western blotting (B) showed a higher expression of SLC45A4 in TP53 mutant cell lines compared to TP53 wild‐type cell lines. Wt, wild‐type; Mut, mutation. Knockdown of SLC45A4 was confirmed by qRT‐PCR (C) and western blotting (D). MTS (E), colony formation (F) and EdU incorporation (G) assays were performed with si‐SLC45A4 or control cells in hTERT‐HPNE, HPAF‐II and MIA PaCa‐2 cell lines. Scale bar = 200 μm. The data are presented as the mean ± SD. *p < 0.05, **p < 0.01 and ***p < 0.001

To further investigate the biological role of SLC45A4 in PDA, we detected the mRNA and protein expression of SLC45A4 in PDA cell lines and the hTERT‐immortalized human pancreatic duct cell line hTERT‐HPNE. The majority of TP53 mutant cell lines exhibited a higher expression of SLC45A4 compared to TP53 wild‐type cell hTERT‐HPNE, Capan2 and SW1990. HPAF‐II harbors the TP53 P151S mutation and MIA PaCa‐2 harbors the TP53 R248W mutation, which are two oncogenic mutations. Interestingly, HPAF‐II and MIA PaCa‐2 showed higher expression of SLC45A4 (Figure 2A, B). Thus, we chose HPAF‐II and MIA PaCa‐2 cells for further study, using hTERT‐HPNE cells as a control. We knocked down SLC45A4 by two siRNAs (Figure 2C, D). The MTS assay showed that SLC45A4 knockdown significantly inhibited the growth of HPAF‐II and MIA‐PaCa‐2 cells, but not hTERT‐HPNE cells (Figure 2E), which was in accordance with the colony formation assays (Figure 2F) and EdU staining (Figure 2G). These findings suggested that the suppression of SLC45A4 expression inhibited proliferation in TP53 mutant PDA cells *in vitro*.

**FIGURE 3:**
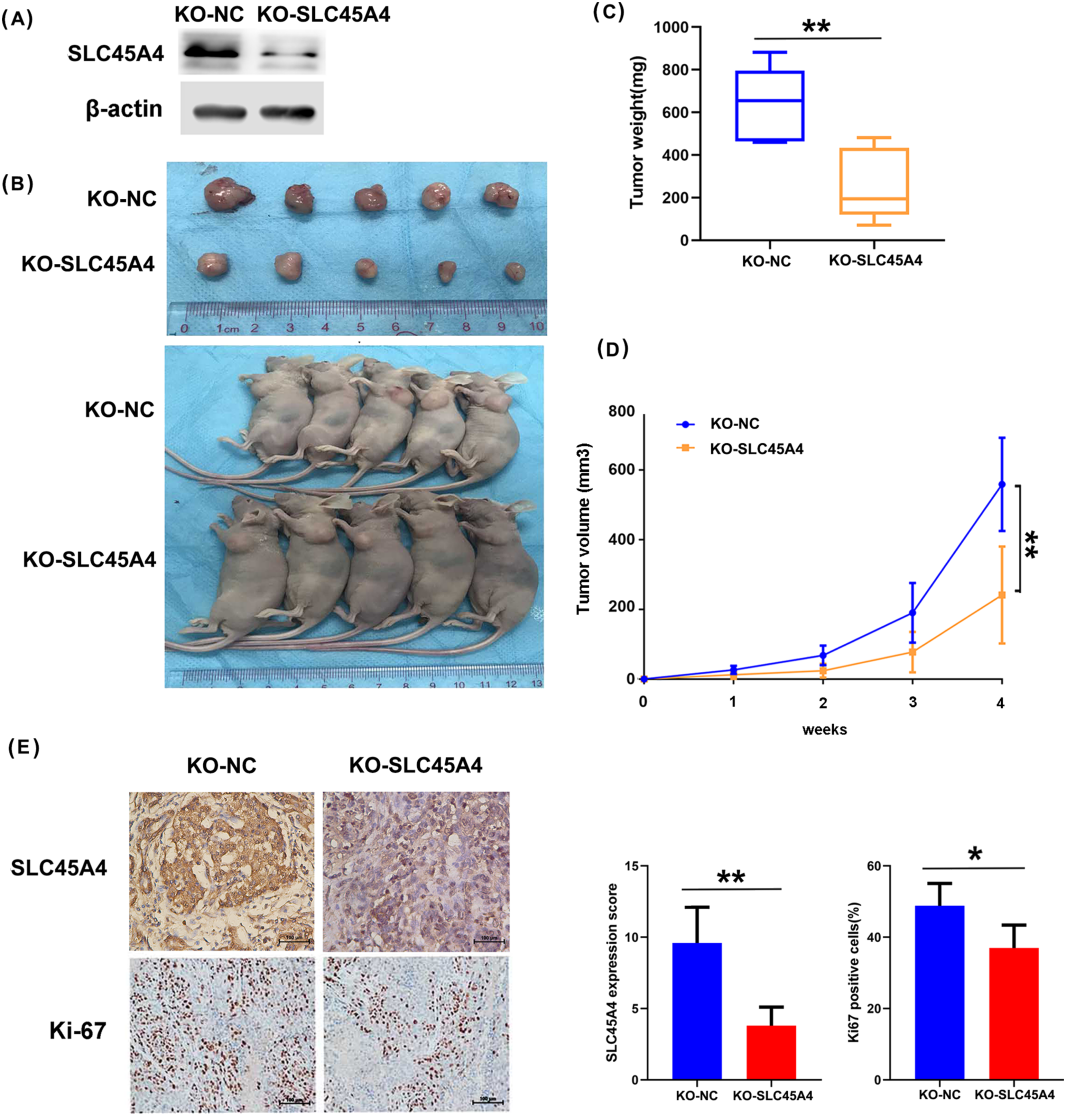
Knockout of SLC45A4 suppresses TP53 mutant PDA cell growth *in vivo*. (A) Knockout of SLC45A4 was detected by western blotting. (B) Knockout of SLC45A4 in HPAF‐II cell inhibited tumor growth *in vivo* (*n* = 5 per group). (C) Tumor weights and (D) growth curves of xenograft tumors after subcutaneous injection with KO‐SLC45A4 HPAF‐II cell or the control cell. (E) Representative images are shown of immunohistochemical staining of SLC45A4 and Ki67 with statistical analysis. Data are presented as the mean ± SD (*n* = 5). *p < 0.05, **p < 0.01 and ***p < 0.001

Furthermore, we knocked out SLC45A4 using the CRISPR/Cas9 system in HPAF‐II cells (Figure 3A) and established xenografts using these cells in nude mice. Tumor growth and weight in the SLC45A4 silencing group were repressed (Figure 3B–D). Moreover, decreased expression of Ki‐67 and SLC45A4 was observed in the SLC45A4 silencing group (Figure 3E). Taken together, these results indicated that suppression of SLC45A4 expression attenuated proliferation potential in TP53 mutant PDA cells.

### 3.3 Knockdown of SLC45A4 expression decreased glucose utilization and ATP production in TP53 mutant PDA cells

**FIGURE 4:**
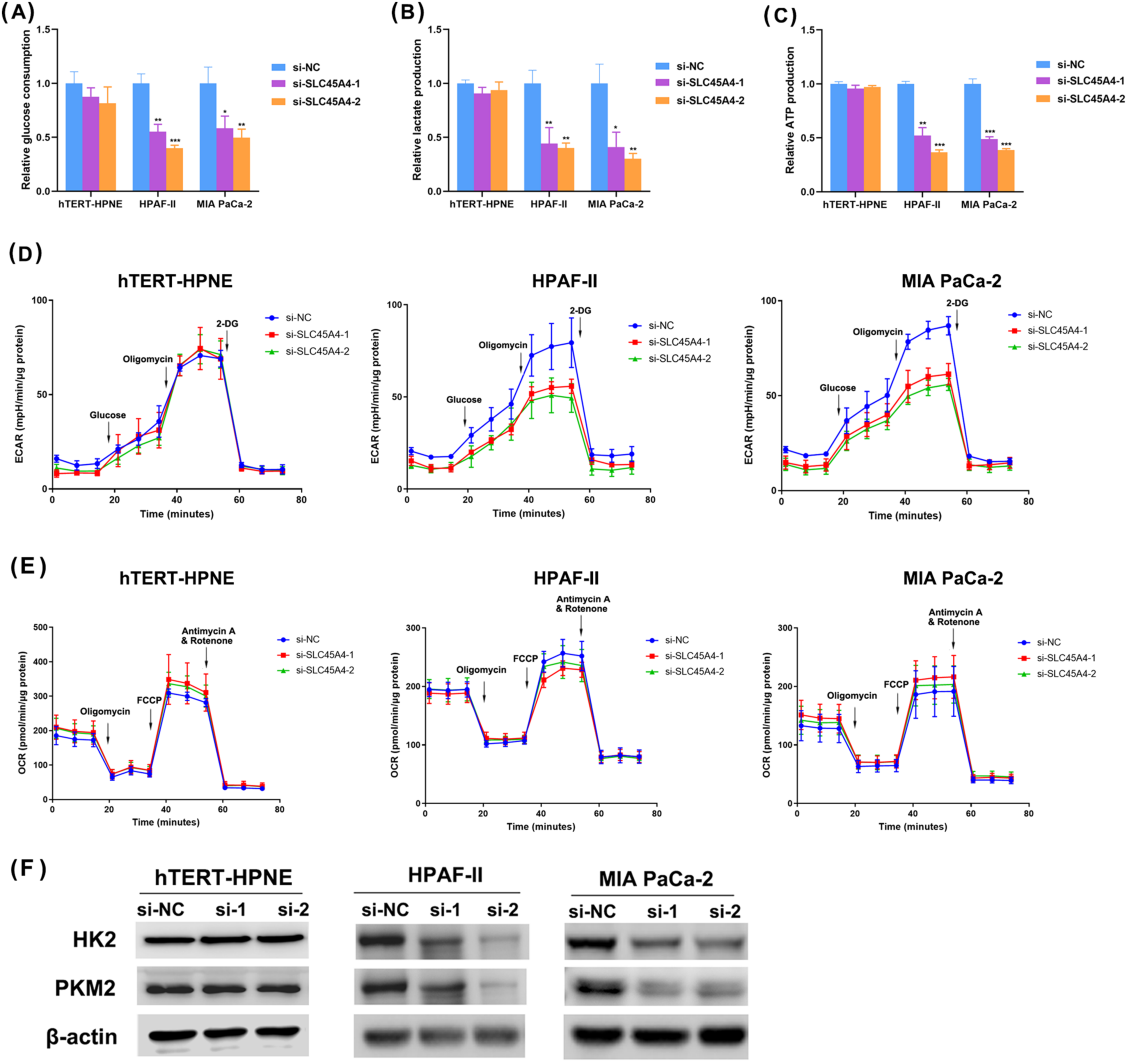
Suppression of SLC45A4 expression reduces glucose utilization and ATP production in TP53 mutant PDA cells. Glucose consumption (A), lactate production (B) and ATP production (C) decreased after SLC45A4 knockdown in HPAF‐II and MIA PaCa‐2 cells, but not in hTERT‐HPNE cell. (D) SLC45A4 knockdown reduced the glycolytic activity and glycolytic capacity in HPAF‐II and MIA PaCa‐2 cells, but not in hTERT‐HPNE cells. (E) No significant difference between SLC45A4 knockdown cells and control cells in OCR. (F) SLC45A4 knockdown decreased HK2 and PKM‐2 protein expression levels in HPAF‐II and MIA PaCa‐2 cells, but not in hTERT‐HPNE cells. Data are presented as the mean ± SD. *p < 0.05, **p < 0.01 and ***p < 0.001

Next, we explored how SLC45A4 promoted proliferation of TP53 mutant PDA cells. Based on its biological function, and considering the biological function of SLC45A4 as a H^+^/sugar symporter, we considered whether it exerted its function through reprogramming glucose metabolism. In TP53 mutant HPAF‐II and MIA PaCa‐2 cells, but not in hTERT‐HPNE cells, SLC45A4 knockdown induced markedly decreased glucose consumption (Figure 4A), lactate production (Figure 4B) and ATP production (Figure 4C). Furthermore, we measured glycolysis by analysing the ECAR and measured mitochondrial oxidative phosphorylation on the basis of the OCR. We found that SLC45A4 knockdown significantly reduced the glycolytic activity and glycolytic capacity (Figure 4D). However, there was no significant difference in OCR between SLC45A4 knockdown and control cells, indicating that SLC45A4 knockdown does not affect oxidative phosphorylation (Figure 4E). HK2 and PKM2 play a critical role in aerobic glycolysis. Intriguingly, SLC45A4 knockdown also down‐regulated the level of HK2 and PKM2 proteins in HPAF‐II and MIA PaCa‐2 cells (Figure 4F). These results revealed that inhibition of SLC45A4 expression repressed glucose utilization and ATP production in TP53 mutant PDA cells.

### 3.4 Down‐regulation of SLC45A4 induced autophagy through the AMPK/ULK1 pathway

**FIGURE 5:**
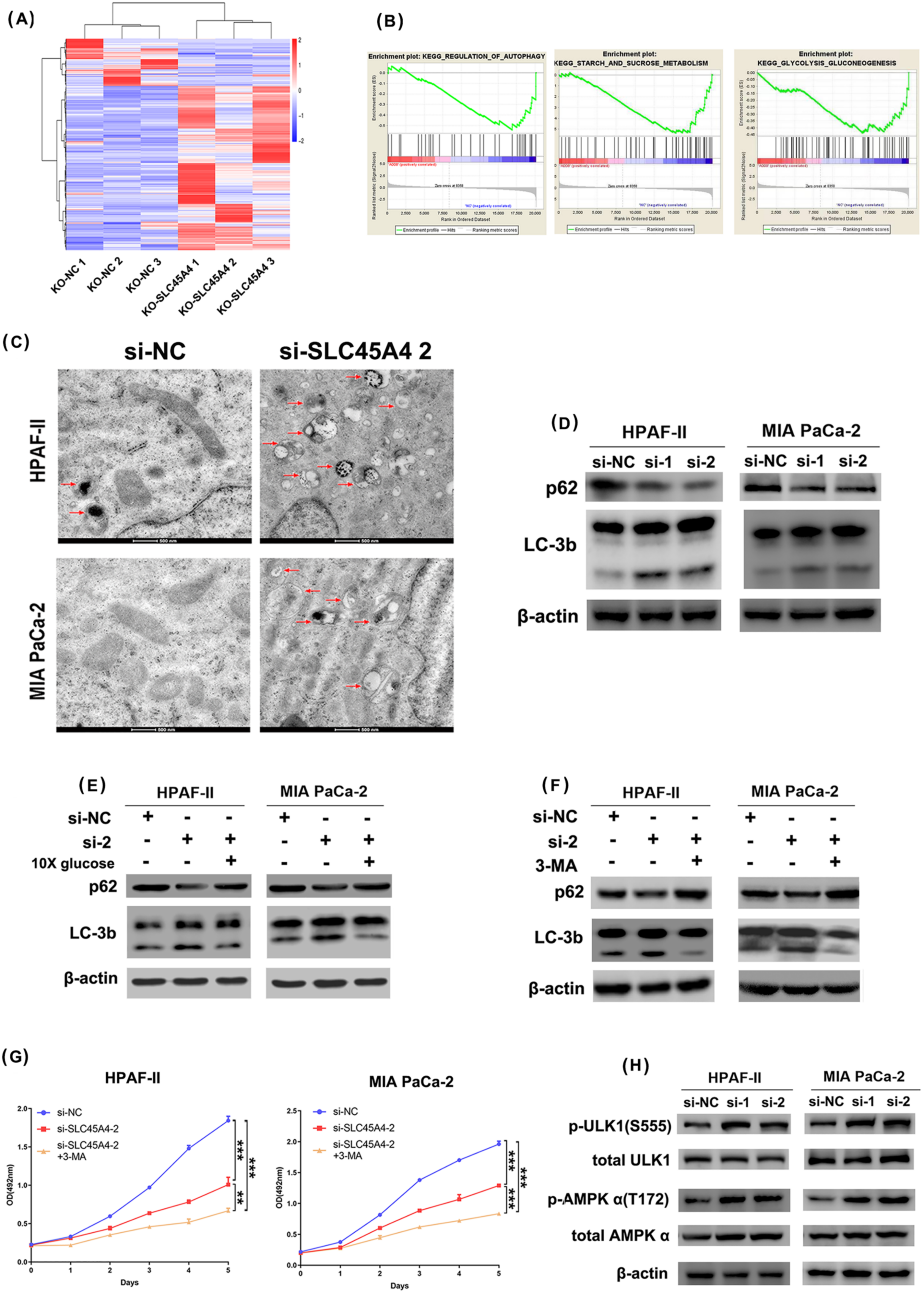
Inhibition of SLC45A4 activates autophagy through the AMPK/ULK1 pathway. (A) Hierarchical clustering heat maps of RNA‐seq data depict the changes in gene expression between HPAF‐II KO‐SLC45A4 and control cells. For each group, triple biological repeats were analyzed. (B) GSEA revealed that autophagy, starch and sucrose metabolism and glycolysis process gene set were affected by SLC45A4 knockout. (C) TEM showing autophagosome/autolysosome of cells with or without SLC45A4 knockdown in HPAF‐II and MIA PaCa‐2 cells. Scale bar = 500 nm. (D) Western blot analysis of p62 and LC3b in HPAF‐II and MIA PaCa‐2 cells with or without SLC45A4 knockdown. (E) Activation of autophagy in HPAF‐II and MIA PaCa‐2 cells with SLC45A4 knockdown can be reversed by excessive glucose refeeding, as measured by western blotting of autophagy markers LC3b and p62. (F) Activation of autophagy in HPAF‐II and MIA PaCa‐2 cells with SLC45A4 knockdown can be inhibited by 3‐MA. (G) A MTS assay showed that 3‐MA intensified the cell growth inhibition caused by SLC45A4 knockdown in HPAF‐II and MIA PaCa‐2 cells. (H) Western blot analysis of total and phosphorylated ULK1/AMPK in HPAF‐II and MIA PaCa‐2 cells with or without SLC45A4 knockdown

To further investigate the biological alteration caused by CRISPR/Cas9‐induced SLC45A4 knockout, we employed RNA‐seq and hierarchical clustering analysis in SLC45A4 deleted and NC HPAF‐II cells (Figure 5A). GSEA showed that SLC45A4 was associated with the autophagy gene set [normalized enrichment score (NES) = −1.68, *p* < 0.001, FDR = 0.10], starch and sucrose metabolism (NES = −1.54, *p* < 0.001, FDR = 0.218) and the glycolysis process gene set (NES = −1.55, *p* < 0.001, FDR = 0.25) in HPAF‐II cells (Figure 5B). TEM analysis confirmed that knockdown of SLC45A4 led to autophagosome/autolysosome accumulation in HPAF‐II and MIA PaCa‐2 cells (Figure 5C). In addition, increased expression of the autophagosomal marker LC3b II and decreased expression of p62, a marker of autophagy inhibition, were observed in SLC45A4 knockdown cells (Figure 5D). Glucose refeeding after SLC45A4 knockdown in TP53 mutant PDA cells reversed the activation of autophagy caused by SLC45A4 knockdown (Figure 5E). Moreover, inhibition of autophagy with 3‐methyladenine (3‐MA) intensified the cell growth inhibition caused by SLC45A4 knockdown in TP53 mutant PDA cells (Figure 5F, G). These results revealed that the activation of autophagy caused by glucose limitation supported cell proliferation.

Autophagy has been reported to be induced by the energy sensing AMPK/ULK1 pathway.^19^, ^20^ As mentioned above, we found that ATP production decreased in SLC45A4 silencing cells (Figure 4C). We proposed that knockdown of SLC45A4 might have activated AMPK/ULK1 signaling. Indeed, phosphorylation of AMPK and ULK1 increased after SLC45A4 knockdown in HPAF‐II and MIA PaCa‐2 cells (Figure 5H). Conclusively, these results indicated that silencing SLC45A4 caused energy stress and activated AMPK/ULK1 to induce autophagy in TP53 mutant PDA cells.

## 4 DISCUSSION

In the present study, we found that expression of SLC45A4 was significantly up‐regulated and played an oncogenic role in PDA, especially in TP53 mutant PDA cells and tissues. Furthermore, SLC45A4 acted as an essential glucose transporter, and its knockdown induced autophagy in the TP53 mutant PDA cells; thus, it may serve as a novel marker and potential therapeutic target for TP53 mutant PDA.

The role of glucose metabolic reprogramming in cancer has become increasingly recognized.^21^, ^22^ Recent studies have showed that some cancer cell lines tend to rely on glycose instead of other nutriments for energy supply.^23^ These cells exhibit rapid glucose consumption and lactate secretion. Oncogenic TP53 mutation promotes glucose metabolism in many types of cancer.^7^, ^24^, ^25^ Thus, TP53 mutant PDA cells may be more sensitive to glucose uptake restriction than wild‐type cells. However, the mechanism of how mutant p53 reprograms glucose metabolism as a novel GOF is not fully understood. In the present study, we found that glucose transporter SLC45A4 was highly expressed in TP53 mutant PDAC cells but not in TP53 wild‐type cells. Knocking down SLC45A4 can significantly inhibit the proliferation of TP53 mutant PDAC cells. Thus, we proposed that the overexpressed SLC45A4 may play an important role in sugar transport of TP53 mutant PDA, which confers a growth advantage to TP53 mutant cells. The SLC45A4 is a member of the sugar/H^+^ symporter family. Previous studies have reported the elevated expression of glucose transporters (such as SLC2 and SLC5 family) in many cancers.^26^, ^27^ The SLC45 family have not been given sufficient attention in tumor metabolism because they were newly identified. We found that SLC45A4 knockdown inhibited glucose utilization, glycolysis and ATP production in TP53 mutant PDA cells. A novel GOF of mutant p53 is promoting GLUT1 translocation to the plasma membrane in both cultured mutant TP53 cells and mutant TP53 knock‐in mice.^7^ However, in most solid tumors, deregulated energy metabolism and insufficient perfusion result in an inadequate glucose supply. Moreover, acidic metabolic products and H^+^ ions tend to accumulate in the tumor microenvironment.^28^, ^29^, ^30^ Because the optimal working pH of the SLC45 family is approximately 6.5,^14^ we proposed that the SLC45A4 may play an important role in sugar transport of TP53 mutant PDA. As the proton‐gradient exists across the plasma membrane, SLC45A4 may provide glucose to cancer cells more efficiently than SLC2 family and SLC5 family.

SLC45A4 knockdown also down‐regulated the level of HK2 and PKM2 proteins. HK2 is the first glycolysis rate‐limiting enzyme that phosphorylates glucose to generate glucose 6‐phosphate.^31^ PKM2 is another key enzyme of glycolysis that catalyzes phosphoenolpyruvate to form pyruvate.^32^ Indeed, Rempel *et al*.^33^ reported that glucose can act as an activator of the HK2 promoter. Moreover, the expression of PKM2 cannot maintained upon hypoglucose.^34^ These studies suggested that the expression of HK2 and PKM2 is associated with the level of intercellular glucose. In the present study, we found that knockdown of SLC45A4 resulted in a decrease of glucose uptake, which led to the down‐regulation of HK2 and PKM2. We presume that the inhibition of SLC45A4 in TP53 mutant PDA cells represses the uptake of glucose and then decreases the intercellular glucose level. Thus, the high level of HK2 and PKM2 protein expression cannot be maintained.

It remains unclear how mutant p53 mechanistically upregulates SLC45A4 expression. Various evidence suggests that mutant p53 proteins can bind to DNA response elements or interact with transcription factors (TF) that are not normally bound by wild‐type p53, enhancing the transcription of target genes.^35^, ^36^ In future work, we aim to determine whether mutant p53 regulates SLC45A4 transcription directly by binding to its promoter or by forming complexes with other TF, or indirectly up‐regulates the SLC45A4 level by other mechanisms.

Recent reports reveal that functional inhibition of sugar transporters is able to modulate the autophagy process *in vitro* and *in vivo*. Empagliflozin, a SGLT2 inhibitor, can rectorate autophagy in cardiomyocytes of the rat model of type 2 diabetes.^37^ Moreover, trehalose, a competitive inhibitor of GLUT transporters, causes a pseudo‐starvation like phenotype, which results in activation of autophagy.^38^ In the present study, GSEA indicated that the autophagy process was significantly affected by SLC45A4 knockdown. Autophagy plays dual roles in tumor promotion and suppression.^39^, ^40^ A basal level of autophagy is necessary for cells to maintain cellular homeostasis. However, when it comes to extremely nutrient deprivation, the role of autophagy is controversial. On the one hand, autophagy provides an alternative energy source for cells to rapidly resist nutritional stress.^41^, ^42^ On the other hand, the amplitude of autophagy increases above a threshold level, leading to a cell death mechanism.^43^, ^44^ The present study revealed that autophagy was induced by SLC45A4 knockdown in TP53 mutant PDA cells. Because inhibition of autophagy with 3‐MA intensified the cell growth inhibition caused by SLC45A4 knockdown in TP53 mutant PDA cells, we can infer that the activation of autophagy caused by glucose limitation supported the cell proliferation.

In summary, the present study reveals that SLC45A4 is highly expressed in TP53 mutant PDA tissues and cell lines. Overexpression of SLC45A4 is associated with tumor progression and the poor prognosis of PDA patients. Knockdown of SLC45A4 inhibits both glucose utilization and ATP production in TP53 mutant PDA cells and activates the AMPK/ULK1 signaling pathway, resulting in the induction of autophagy. The present study provides a better understanding of the H^+^‐dependent sugar cotransporter in TP53 mutant PDA progression and SLC45A4 may be a potential target with respect to developing therapeutic strategies.

## CONFLICT OF INTEREST STATEMENT

The authors declare that they have no conflicts of interest.

## AUTHOR CONTRIBUTIONS

WC, FH and SZ were responsible for the conceptualization and design of the study. WC, FH and YL were responsible for the study methodology. JH, JP, YZ and XH were responsible for study investigations and data curation. WC, FH, ZZ and LL were responsible for the formal analysis and interpretation of data. WC, FH and JH were responsible for drafting or revising the manuscript. SZ was responsible for study supervision and project administration. All authors have read and approved the final version of the manuscript submitted for publication.

## Data Availability

The data that support the findings of this study are available in GEPIA, the Human Protein Atlas, cBioporta and the CCLE. These data were derived from the following resources available in the public domain (http://gepia.cancer-pku.cn/index.html, https://www.proteinatlas.org/, https://www.cbioportal.org/, https://portals.broadinstitute.org/ccle). Other data that support the findings of this study are available from the corresponding author upon reasonable request.
